# Comparative Analysis of Pericapsular Nerve Group (PENG) Block Versus Femoral Nerve Block for Postoperative Pain Management in Total Hip Arthroplasty: An Observational Prospective Study

**DOI:** 10.7759/cureus.84887

**Published:** 2025-05-27

**Authors:** Luís Gonçalves, Ana Santos, Catia Domingues, Elisabete Valente, Lucia Gonçalves, Marta Laranjo, Alexandra Lagarto, Elisa Ferreira, Vasco Simões

**Affiliations:** 1 Anesthesiology, Centro Hospitalar de Leiria, Leiria, PRT; 2 Anesthesiology, Unidade Local de Saúde da Região de Leiria, Leiria, PRT; 3 Physical Medicine and Rehabilitation, Unidade Local de Saúde da Região de Leiria, Leiria, PRT

**Keywords:** femoral nerve, pain management, postoperative pain, regional anesthesia, total hip arthroplasty

## Abstract

Introduction: Total hip arthroplasty (THA) improves patient mobility and quality of life but is also associated with postoperative pain. Effective pain management is crucial for early postoperative mobility and optimal recovery. Regional analgesia techniques such as femoral nerve block (FNB) are commonly used, but recent studies suggest the pericapsular nerve group (PENG) block may provide superior hip analgesia with less motor impairment.

Objectives: This study compares the effectiveness of PENG block versus FNB concerning postoperative analgesia and motor block sparing in THA patients.

Materials and methods: An observational prospective study was conducted at the Unidade Local de Saúde da Região de Leiria, with data collected from January 2023 to April 2024. Patients aged 18 years or older undergoing primary THA and receiving either a PENG block or an FNB were included.

Results: A total of 55 patients (17 in the FNB group and 38 in the PENG group) participated in this study. Both blocks effectively reduced postoperative pain scores with no significant differences between groups, demonstrating their efficacy in pain management. Postoperative knee extension motor scores were significantly higher in the PENG group compared to the FNB group (1.52 vs. 1.17, p=0.036), indicating better preservation of motor function. Similarly, leg adduction motor scores were higher in the PENG group than in the FNB group (1.50 vs. 1.29, p=0.02).

Conclusion: The PENG block demonstrated superior motor function preservation and longer analgesia following THA compared to the FNB. The PENG block may offer a clinical advantage in facilitating earlier mobilization and improving overall recovery outcomes post-surgery.

## Introduction

Total hip arthroplasty (THA) is a surgical procedure performed with the aim of improving the mobility and quality of life of patients suffering from hip pain [1]. THA is indicated for patients who present deterioration of the hip joint and for whom conservative treatment has not proven sufficient. Surgical conditions that may require THA include, but are not limited to, osteoarthritis, inflammatory diseases (such as rheumatoid arthritis and psoriatic arthritis), femoro-acetabular impingement, developmental dysplasia of the hip, trauma, tumors, and osteonecrosis. Due to the progressive aging of the population, along with the risk factors associated with these conditions, surgery is increasingly becoming a necessity [2].

THA is usually followed by moderate to severe pain. Effective pain management includes conventional analgesia, use of opioids, and regional anesthesia [3,4]. Adequate analgesia with minimal safety risks is essential for early postoperative mobility, optimal functional recovery, and reduction of postoperative morbidity [1,3,4]. Regional analgesia techniques, such as femoral nerve block (FNB), fascia iliaca block (FIB), and three-in-one FNB, are popular due to their opioid-sparing effects and reduced safety risks associated with their administration. However, their analgesic impact is only moderate, and studies suggest that the obturator nerve (ON) is not covered or not consistently covered by these techniques [3,4]. The femoral nerve innervates the quadriceps femoris (responsible for leg extension), while the ON innervates the leg adductors, which are responsible for leg adduction. The anterior capsule of the hip is innervated by the ON, the accessory obturator nerve (AON), and the femoral nerve (the most innervated part of the joint), suggesting that these nerves should be the main targets for hip analgesia [3,5]. FNB blocks only the femoral nerve, providing hip analgesia but causing motor blockade of the quadriceps femoris and the pectineus and sartorius muscles, making hip flexion and knee extension difficult.

On the other hand, the pericapsular nerve group (PENG) block blocks the high articular branches of the AON, the femoral nerve, and the ON in the region between the anterior inferior iliac spine and the ilio-pubic eminence, sparing the motor branches of the femoral nerve [3,4,6-8].

To date, no clinical studies have been carried out in Portugal to compare the efficacy and safety of the PENG block with the FNB. This is the first study conducted in this country to assess these techniques in a real-world clinical setting. The primary objective of this study was to compare the effectiveness of PENG block with FNB in THA regarding postoperative analgesia and motor block sparing. Secondary objectives included characterizing the efficacy, safety, and patient satisfaction with PENG block and analyzing the perioperative course of patients undergoing THA, considering the quality of postoperative analgesia (amount of on-demand analgesia performed and/or use of opioids), time to ambulation, and length of hospital stay.

## Materials and methods

This observational prospective study was carried out at the Unidade Local de Saúde da Região de Leiria (ULSRL) in Portugal from January 2023 to April 2024 and was approved by the site ethics committee. Patients indicated for THA were invited to participate in the study and asked to sign the informed consent before performing the peripheral nerve block (PNB) technique. The technique, which was selected according to the anesthesiologist's clinical judgment, was performed using 15-20 ml of 0.5% ropivacaine at least 45 minutes before the surgery was initiated. Total opioid consumption was calculated in morphine milligram equivalents (MME) to standardize the comparison. Tramadol doses were converted to intravenous morphine equivalents using the standard conversion ratio (10 mg tramadol = 1 mg intravenous morphine). Oral morphine was converted to intravenous morphine equivalents using the standard conversion ratio (3 mg oral morphine = 1 mg intravenous morphine).

Patients aged 18 years or older who underwent primary THA and received either a PENG block or an FNB for postoperative analgesia were included. Exclusion criteria included allergy to local anesthetics, infection at the injection site, weight less than 30 kg, ASA (American Society of Anesthesiologists) classification ≥4, dementia or cognitive impairment, contraindications to PNB, and patients who refused or were unable to consent to participation. Data collected included patient demographics and relevant baseline characteristics, information characterizing the surgical and anesthetic procedures, and outcomes and complications during the postoperative and recovery period. This study did not collect any potentially identifying data. Data were recorded on an electronic form specific to this study, which served as the basis for the statistical analysis of the results.

The primary outcome measure was the effectiveness of postoperative analgesia and motor block sparing. This was assessed by evaluating pain scores and motor function at three time points: before the nerve block, 30 minutes after, and postoperatively. Pain was assessed using the Numeric Rating Scale (NRS), where 0 indicated no pain and 10 indicated the worst possible pain. Motor function evaluation of the hip flexors, quadriceps femoris, and hip adductors was conducted using a score of 0 (no motor function), 1 (reduced motor function), and 2 (normal motor function), compared to the contralateral limb.

Secondary outcomes included total opioid consumption, which was measured in MME during the PACU (Post-Anesthesia Care Unit) stay, after PACU, and for the total postoperative period. The incidence of complications, including PNB-related complications, medical complications (e.g., hypotension, nausea, vomiting, anemia requiring transfusion, atrial fibrillation, extreme bradycardia), and surgical complications; patient satisfaction, which was assessed using a scale from 0 to 5; time to ambulation, which was recorded to assess the impact of the nerve block on mobility; and total duration of hospital stay in days. Patients were assessed before and after 30 minutes of the PNB technique and in the postoperative period.

Data collected included pain scores, motor function, and sensory assessments. Sensory assessments involved the medial and lateral portions of the anterior thigh. In the recovery period, the need for unplanned analgesia and any possible intercurrences were recorded. Postoperative data were collected 12 to 24 hours after surgery. Additionally, the incidence of postoperative complications, the time when the nerve block effects were perceived to have worn off, and patient satisfaction levels were recorded. Follow-up data included total opioid consumption during hospitalization, total length of hospital stay from surgery to discharge, time to first ambulation measured from the end of surgery to the first instance of ambulation, and the occurrence of any adverse events during the postoperative period.

A formal sample size was not calculated for this study. All eligible and consenting patients during the study period were included in the study. Descriptive statistics included means and standard deviations (SD) for normally distributed continuous variables, medians and quartiles (Q1-Q3) for non-normal distributions, absolute and relative frequencies for categorical variables, and percentages for categorical variables. Normality of distributions was assessed using the Shapiro-Wilk test. The primary analysis used repeated measures ANOVAs (RM ANOVAs) to evaluate changes in pain, femoral sensory, obturator sensory, knee extension motor, and leg adduction motor scores before the nerve block, 30 minutes after, and postoperatively. RM ANOVAs tested the main and interaction effects of time and nerve block type (FNB vs. PENG). Post hoc pairwise comparisons with Sidak correction addressed multiple testing. Results included F-statistics, p-values, and partial eta squared (η²ₚ) for effect size (small: 0.01, moderate: 0.06, large: 0.14). The assumptions checked were residual normality, sphericity (Mauchly's test), and homogeneity of variances (Levene's test), all of which met with p-values > 0.05. The null hypothesis was rejected at p < 0.05. Data were analyzed using IBM SPSS Statistics for Windows, Version 29 (Released 2023; IBM Corp., Armonk, New York).

## Results

Table 1 presents the demographic and baseline characteristics of the 55 patients in the study, who were divided into the FNB and PENG groups (17 in the FNB group and 38 in the PENG group). Both groups were similar regarding mean age, sex, and mean BMI. The median ASA classification was 2.0 in both groups. The most common surgical indication was hip osteoarthritis (88.2% in the FNB group and 100.0% in the PENG group).

**Table 1:** Demographic and baseline characteristics of the patients Means and standard deviations (SD) were used for normally distributed variables, while medians and interquartile ranges (IQR) were used for variables with non-normal distributions. The p-values were computed using (a) t-tests, (b) Fisher tests, and (c) Mann-Whitney tests. FNB: femoral nerve block group; PENG: pericapsular nerve group; BMI: body mass index; IQR: interquartile range; SD: standard deviation

Variable	Total (n=55)	FNB (n=17)	PENG (n=38)	p-value
Age (years), mean (SD)	67.8 (12.8)	66.8 (15.5)	68.8 (10.5)	t_(53)_= -0.57, p=0.573 (a)
Sex, n (%)				
Male	27 (49.1%)	9 (52.9%)	18 (47.4%)	p=0.706 (b)
Female	28 (50.9%)	8 (47.1%)	20 (52.6%)	
BMI, mean (SD)	27.7 (4.4)	27.4 (5.4)	27.9 (3.8)	t_(53)_= -0.40, p=0.694 (a)
ASA classification, median (IQR)	2.0 (2.0 - 3.0)	2.0 (2.0 - 3.0)	2.0 (2.0 - 3.0)	Z=-0.89, 0.374 (c)
Prior opioid use, n (%)				
No	51 (92.7%)	15 (88.2%)	36 (94.7%)	p=0.580 (b)
Yes	4 (7.3%)	2 (11.8%)	2 (5.3%)	
Surgical indication, n (%)				
Hip osteoarthritis	53 (96.3%)	15 (88.2%)	38 (100.0%)	p=0.092 (b)
Avascular necrosis	1 (1.8%)	1 (5.9%)	0 (0.0%)	
Legg-Calvé-Perthes disease	1 (1.8%)	1 (5.9%)	0 (0.0%)	

The lateral surgical approach was predominantly used across both groups, and most patients received a subarachnoid block (SAB) as the anesthetic plan. Complications related to the PNB were minimal, with only one case of pain reported in each group during block administration. The most common medical complication observed in both groups was hypotension, occurring more frequently in the FNB group (35.3%) compared to the PENG group (15.8%). This suggests a higher risk of hypotension in patients receiving the FNB. Additionally, there were a few more severe cases in the FNB group, including one instance of atrial fibrillation and one case of extreme bradycardia. However, all complications were managed successfully and resolved within expected clinical outcomes, with no significant deviations from anticipated recovery processes.

Prior opioid use was low in both groups, with no significant differences observed. Although both groups showed variability in the timing of pain relief and mobilization, no clinically significant differences were observed between the FNB and PENG groups. Most patients in both groups received their first dose of pain relief within 24-48 hours postoperatively, and most patients were able to mobilize within the same time frame. The duration of the nerve block and patient satisfaction ratings were also similar between the groups, with no significant differences noted. For postoperative pain relief during the PACU stay, tramadol was used by one patient (5.9%) in the FNB group and five patients (13.2%) in the PENG group; morphine was administered to three patients (17.6%) in the FNB group and three patients (7.9%) in the PENG group. After PACU stay, 64.7% of patients in the FNB group and 47.4% in the PENG group used tramadol, while no patient required morphine in either group. Table 2 summarizes the surgical characteristics and postoperative outcomes by block type.

**Table 2:** Surgical characteristics and postoperative outcomes by block type Means and standard deviations (SD) were used for normally distributed variables, while medians and interquartile ranges (IQR) were used for variables with non-normal distributions. The p-values were computed using (a) t-tests, (b) Fisher's tests, and (c) Mann-Whitney tests. Opioid doses were converted to oral morphine equivalents (OME) using standard conversion ratios. FNB: femoral nerve block group; PENG: pericapsular nerve group; BMI: body mass index; IQR: interquartile range; SD: standard deviation

Variable (N=55)	FNB (n=17)	PENG (n=38)	p-value
Length of stay (days), mean (SD)	3.5 (0.7)	3.2 (0.9)	t_(53)_= -0.57, p=0.194 (a)
Surgical approach, n (%)			
Anterior	2 (11.8%)	1 (2.6%)	p=0.223 (b)
Lateral	15 (88.2%)	37 (97.4%)	
Anesthetic plan, n (%)			
Subarachnoid block	15 (88.2%)	31 (81.6%)	p=0.705 (b)
General anesthesia	2 (11.8%)	7 (18.4%)	
PNB-related complications, n (%)			
None	16 (94.1%)	37 (97.4%)	p=0.527 (b)
Pain	1 (5.9%)	1 (2.6%)	
Medical complications, n (%)			
None	7 (41.2%)	28 (73.7%)	-
Hypotension	6 (35.3%)	6 (15.8%)	
Nausea and vomiting	2 (11.8%)	2 (5.3%)	
Anemia requiring transfusion	0 (0.0%)	2 (5.3%)	
Atrial fibrillation	1 (5.9%)	0 (0.0%)	
Extreme bradycardia	1 (5.9%)	0 (0.0%)	
Surgical complications, n (%)			
No	17 (100.0%)	36 (94.7%)	p>0.990 (b)
Yes	0 (0.0%)	2 (5.3%)	
Mobilization time, n (%)			
< 24 h	0 (0.0%)	0 (0.0%)	p=0.407 (b)
24–48 h	11 (73.3%)	17 (77.3%)	
48–72 h	4 (26.7%)	3 (13.6%)	
72–96 h	0 (0.0%)	2 (9.1%)	
Not assessed (excluded from %)	2	16	
Block end time, n (%)			
< 6 h	3 (17.6%)	6 (16.2%)	-
6–12 h	9 (52.9%)	17 (45.9%)	
12–24 h	4 (23.5%)	6 (16.2%)	
Don't know	1 (5.9%)	8 (21.6%)	
Not assessed (excluded from %)	0	1	
Patient satisfaction rating (0–5), median (IQR)	5.0 (4.0 - 5.0)	5.0 (4.0 - 5.0)	Z=-0.49, p=0.625 (c)
Tramadol use in the PACU, n (%)			
No	16 (94.1%)	33 (86.8%)	-
Yes	1 (5.9%)	5 (13.2%)	
Morphine use in the PACU (mg), n (%)			
No	14 (82.4%)	35 (92.1%)	-
Yes	3 (17.6%)	3 (7.9%)	
Tramadol use after PACU (mg), n (%)			
No	6 (35.3%)	20 (52.6%)	-
Yes	11 (64.7%)	18 (47.4%)	
Morphine use after PACU (mg), n (%)			
No	17 (100.0%)	38 (100.0%)	-
Yes	0 (0.0%)	0 (0.0%)	
Amount of IV morphine equivalent use in the PACU (mg), median (IQR)	0.00 (0.00 - 0.00)	0.00 (0.00 - 0.00)	Z=-0.09, p=0.930 (c)
Amount of IV morphine equivalent use after PACU (mg), median (IQR)	3.33 (0.00 - 31.67)	0.00 (0.00 - 3.33)	Z=-1.84, p=0.066 (c)
Amount of IV morphine equivalent use in the total postoperative period (mg), median (IQR)	3.33 (0.00 - 21.78)	3.33 (0.00 - 5.89)	Z=-1.69, p=0.090 (c)
First pain relief dose, n (%)			
No pain relief dose	8 (47.1%)	19 (50.0%)	p=0.674 (b)
< 24 h	6 (35.3%)	13 (34.2%)	
24–48 h	2 (11.8%)	5 (13.2%)	
48–72 h	1 (5.9%)	1 (2.6%)	
72–96 h	0 (0.0%)	0 (0.0%)	
Not assessed (excluded from %)	0	0	

**Figure 1:**
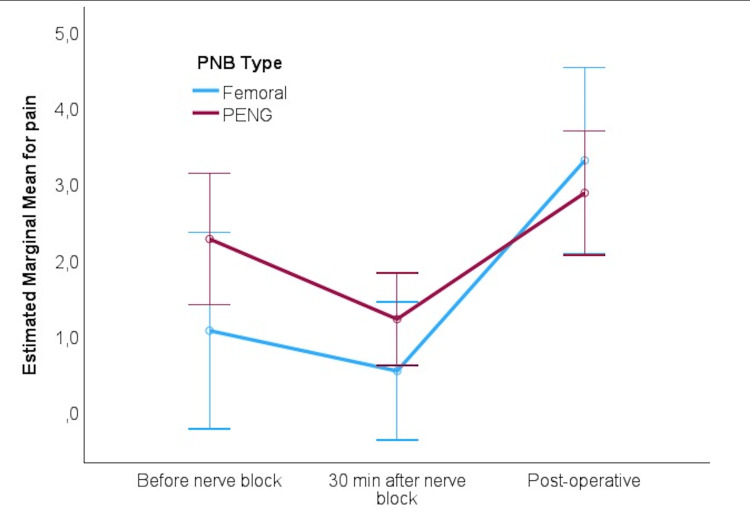
Adjusted marginal means for pain score during the observation period PNB: peripheral nerve block; PENG: pericapsular nerve group

Table 3 presents RM ANOVA results for pain scores measured at three time points: pre-block, 30 minutes post-block, and postoperatively. Both FNB and PENG blocks were effective in significantly reducing pain over time. Although pain scores decreased substantially in both groups, no significant difference was observed between the groups regarding the changes in pain scores (see Figure 1).

**Table 3:** RM ANOVA for pain score Results are presented as adjusted means (adjM) and standard errors (SE); effect size is calculated as partial eta squared (η2p) following Cohen's (1988) guidelines for low (0.01), moderate (0.06), and high (0.14) effect sizes; *p<0.05; **p<0.01; ***p<0.001.

Group	Before nerve block	30 min after	Postoperative	Main effect of nerve block	Nerve x block type effect
Total	1.66 (0.39)	0.87 (0.27)	3.08 (0.37)	F_(2)_=14.43. p<0.001***. η^2^_p_=0.21	F_(2)_=1.99. P=0.142. η^2^_p_=0.04
FNB	1.06 (0.64)	0.53 (0.45)	3.29 (0.61)		
PENG	2.26 (0.43)	1.21 (0.30)	2.87 (0.41)		

**Figure 2:**
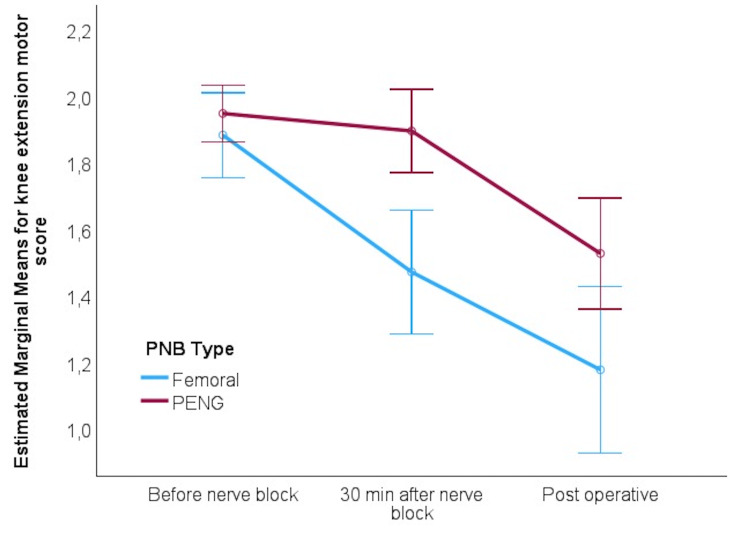
Adjusted marginal means for knee extension motor score during the observation period PNB: peripheral nerve block; PENG: pericapsular nerve group

**Figure 3:**
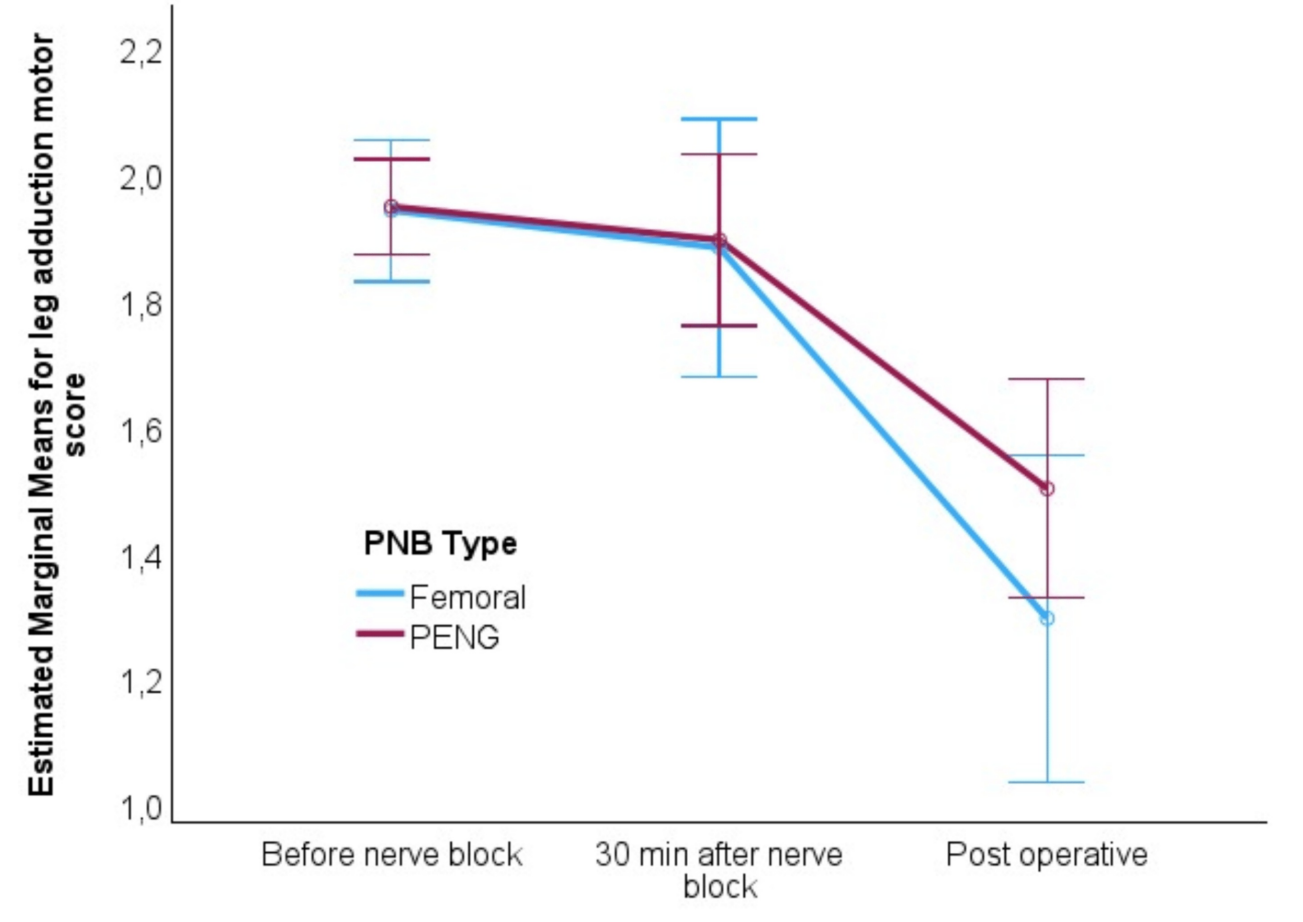
Adjusted marginal means for leg adduction motor score during the observation period PNB: peripheral nerve block; PENG: pericapsular nerve group

The analysis of knee extension and leg adduction motor functions indicated that the PENG block was more effective in preserving motor function compared to the FNB, particularly in the postoperative period. While both blocks led to a decline in motor function over time, the decline was more pronounced in the FNB group for both types of movement, with postoperative scores higher in the PENG group. This motor-sparing advantage of the PENG block is evident in the linear decrease of scores in the FNB group and the delayed reduction in the PENG group, highlighting its superior ability to maintain motor function (Figures 2, 3).

Both the FNB and PENG blocks significantly impacted femoral and obturator sensory scores over time, as indicated by RM ANOVA results (p<0.001). Sensory scores decreased notably 30 minutes after the block compared to pre-block levels, reflecting effective sensory blockade in both nerve territories. However, there were no significant differences between the two groups in terms of sensory score changes, suggesting that the FNB and PENG blocks provided comparable sensory effects across both the femoral and obturator nerves.

## Discussion

This study is the first in Portugal to compare the PENG block with the FNB in THA. Our findings provide insight into the clinical effectiveness of these regional anesthesia techniques, particularly concerning postoperative analgesia and motor function preservation. The primary outcome of this study was the effectiveness of the PENG block versus the FNB in managing postoperative pain. Both techniques significantly reduced postoperative pain scores compared to preoperative levels, underscoring their efficacy in providing analgesia following THA. While the PENG block demonstrated a slightly better performance in maintaining lower postoperative pain scores, this difference was not statistically significant. This finding suggests that while the PENG block may offer some advantages in pain control, both blocks are effective for postoperative analgesia in THA patients.

In comparison with the literature, Lin et al. [7] found that the PENG block provided superior short-term analgesia compared to the FNB, with significantly lower pain scores on the day of surgery and preserved quadriceps strength, which facilitated earlier mobilization. This aligns with our observation that patients receiving the PENG block experienced slightly lower pain scores postoperatively. Similarly, Pascarella et al. [8] reported that the PENG block resulted in better postoperative analgesia and functional recovery than traditional nerve blocks, further supporting our findings of its efficacy in pain management post-THA [8].

In addition to pain scores, opioid consumption was analyzed to evaluate the long-term effectiveness of both blocks in managing postoperative pain. Initially, opioid requirements were minimal for both groups, indicating the immediate efficacy of the regional blocks. However, as patients transitioned from the PACU, a divergence emerged. Patients in the PENG group generally required fewer opioids than those in the FNB group, pointing to more sustained pain relief provided by the PENG block. Although this difference in opioid consumption did not reach statistical significance, it highlights a trend that favors the PENG block in reducing opioid dependency postoperatively. This finding is consistent with results from similar studies [4,7].

Regarding preservation of motor function, the PENG block offered superior motor function preservation compared to the FNB, with significantly higher postoperative knee extension and leg adduction motor scores. This result is important in the context of early mobilization, which is crucial for patient recovery following THA. The PENG block's ability to target the articular branches of the femoral and AON, while sparing the motor branches, likely contributes to this improved motor function. This finding aligns with studies [7-15] that have demonstrated the PENG block's effectiveness in preserving quadriceps strength, thereby facilitating earlier ambulation post-surgery.

It is important to address the safety profile of the techniques. The adverse events observed in this study were consistent with those generally expected for regional anesthesia methods, as reported in other studies. Hypotension was more frequent in the FNB group, although there remains a notable gap in the literature on this specific comparison. While there may be a potential safety advantage to using the PENG block, especially regarding hypotension, it is crucial to approach this conclusion with caution, as the small sample size in the femoral block group could have introduced bias.

One notable limitation of this study is the unequal sample size between the PENG and FNB groups, which may introduce bias and affect the reliability of our findings, even though both groups presented similar demographic characteristics. Additionally, as a single-center observational study, the results may not be generalizable to other settings. The lack of randomization and potential variability in technique due to different practitioners also limit the internal validity of the study. However, our observational approach captures the complexities of real-world patient care, enhancing the applicability of our findings to routine practice. Despite these challenges, the study highlights the PENG block's advantages in motor function preservation, extended analgesia, and fewer complications compared to the femoral block, underscoring its potential value in clinical practice.

## Conclusions

This observational study indicates that both the PENG block and FNB effectively reduce postoperative pain following THA; however, the PENG block demonstrated superior preservation of motor function, which may contribute to enhanced early mobilization and functional recovery. In addition, the trend toward reduced opioid consumption observed in the PENG group further supports its clinical benefits in managing postoperative pain.

Overall, the study provides valuable insights into optimizing regional anesthesia techniques in THA. By balancing effective pain management with motor-sparing properties, the PENG block may improve patient outcomes and facilitate a smoother and quicker recovery process.
